# CPAP Treatment Partly Normalizes Sleep Spindle Features in Obstructive Sleep Apnea

**DOI:** 10.1155/2017/2962479

**Published:** 2017-02-02

**Authors:** Tiia Saunamäki, Eero Huupponen, Juho Loponen, Sari-Leena Himanen

**Affiliations:** ^1^Department of Neurology and Rehabilitation, Tampere University Hospital, Tampere, Finland; ^2^Department of Clinical Neurophysiology, Medical Imaging Centre and Hospital Pharmacy, Pirkanmaa Hospital District, Tampere, Finland; ^3^Faculty of Medicine and Life Sciences, University of Tampere, Tampere, Finland

## Abstract

*Objective*. Obstructive sleep apnea (OSA) decreases sleep spindle density and frequency. We evaluated the effects of continuous positive airway pressure (CPAP) treatment on different features of sleep spindles.* Methods*. Twenty OSA patients underwent two night polysomnographies in a diagnostic phase and one night polysomnography after 6 months of CPAP treatment. The control group comprised 20 healthy controls. Sleep spindles were analyzed by a previously developed automated method. Unilateral and bilateral spindles were identified in central and frontopolar brain locations. Spindle density and frequency were determined for the first and last half of the NREM time.* Results*. The density of bilateral central spindles, which did not change in the untreated OSA patients, increased towards the morning hours during CPAP treatment and in the controls. Central spindles did not become faster with sleep in OSA patients and the central spindles remained slow in the left hemisphere even with CPAP.* Conclusion*. CPAP treatment normalized spindle features only partially. The changes may be associated with deficits in thalamocortical spindle generating loops.* Significance*. This study shows that some sleep spindle changes persist after CPAP treatment in OSA patients. The association of these changes to daytime symptoms in OSA patients needs to be further evaluated.

## 1. Introduction

By standard definition, sleep spindles are composed of transient electroencephalography (EEG) oscillations in the frequency range of 11 Hz to 16 Hz lasting at least 0.5 s [1]. In healthy subjects, faster spindles or spindle activity occur more often in the parietal area, while slower spindles are more prevalent in the frontal regions [2–8]. The intraspindle frequency increases during the morning hours [2, 9]. Particularly the central spindles occur in periodic sequences which are found to be more frequent at the morning hours [10].

Spindles are abundant in light non-REM (NREM) sleep and are supposed to prevent arousing stimuli from reaching the cortex [11], thus ensuring solid sleep [2]. More recently, spindles have been found to play an important role in many brain functions such as in learning and memory processes [12]. These functions seem to be dependent on the long-term potentiation (LTP) induced by the spindle bursts [13]. In particular, the fast spindles (usually >13 Hz) have been found to have an important role in connecting the hippocampus and the neocortex and in transferring temporary hippocampal memories to the neocortex for storage [14, 15]. In addition, local spindles have been presented to arise in cortical areas that were active in motor or visuomotor tasks during preceding wakefulness [16, 17].

Obstructive sleep apnea (OSA) is characterized by repetitive episodes of upper airway obstruction during sleep and results in oxygen desaturation and arousals from sleep. In OSA patients, spindle density decreases, the percentage of slow spindles increases, and the spindles remain slow throughout the night [18–20]. Continuous positive airway pressure (CPAP) treatment is a first-line therapy in moderate and severe OSA. CPAP treatment reduces obstructive breathing events and normalizes oxyhemoglobin saturation and sleep fragmentation. The result of CPAP treatment is improved sleep quality and reduced daytime symptoms. However, some daytime symptoms such as cognitive deficits seem to remain [21, 22]. The effect of CPAP treatment on spindle activity in OSA patients is, however, an open question.

In the present study, it is assumed that CPAP mostly normalizes the disturbed sleep spindle process in OSA but that some impairment in the spindle process may still remain. Since many spindle features change dynamically during nocturnal sleep [9], spindle features are evaluated in the first and last part of the nocturnal NREM sleep.

## 2. Materials and Methods

The study cohort included 20 male OSA patients and 20 age-matched healthy controls. The patients had been referred to the sleep laboratory because of suspected OSA. All patients met the diagnostic criteria of OSA, and their first treatment choice was CPAP. The control subjects were healthy volunteers recruited through advertisements. They were not paid for their participation in the study.

Both patients and controls were first interviewed to ensure they met the initial eligibility criteria: working-aged, right-handed, no (other) sleep disorders, no clinically significant medical disorder (e.g., neurological or psychiatric disease, hypo/hyperthyroidism, or lung disease other than asymptomatic asthma), and no substance or alcohol abuse.

Both study groups underwent a clinical interview and diagnostic polysomnography (PSG) in a sleep laboratory. The OSA diagnosis was based on a clinical picture, subjective complaints and an apnea/hypopnea index (AHI) value of > 10 per hour of sleep. The controls had to be asymptomatic and have an AHI value of < 5 per hour of sleep. The patients and control subjects who, according to the results of the first all-night diagnostic PSG, met the eligibility criteria underwent a second all-night PSG, and the data from this night were used in the analysis.

After 6 months of CPAP treatment, the patients underwent a third PSG. Objective CPAP compliance measures were downloaded from the devices and the minimum required CPAP adherence was set at 4 hours per night and at least 5 nights per week. All patients met the CPAP adherence criteria and were included into the analyses. The study was approved by the Ethical Committee of the Pirkanmaa Hospital District and all subjects gave their written informed consent.

### 2.1. Recordings and Visual Analysis

Subjects went to sleep between 10 pm and 12 pm, according to their own habitual bed times. Six EEG derivations (Fp1-A2, Fp2-A1, C3-A2, C4-A1, O1-A2, and O2-A1), two electrooculography channels, submental muscle tonus, electrocardiogram, airflow pressure by nasal transducer, thermistor, thoracoabdominal respiratory movements, and blood oxygen saturation were recorded. In addition, transcutaneous carbon dioxide tension, leg movements, body position and body movements were also recorded. Polysomnographies were recorded with an Embla® N7000 device (Embla, Natus Medical Inc., USA). The EEG signals were sampled at 200 Hz (16 bits) with a bandwidth of 0.3 Hz to 90 Hz.

As the study began before the revision of the sleep staging rules, the frontopolar EEG channels Fp1 and Fp2 were used instead of the frontal channels F3 and F4 that are recommended nowadays. The sleep staging was, however, performed in accordance with the rules established by the AASM in 2007 [23] with the exception of the EEG channels. Somnologica® software (Medcare/Flaga, Iceland) was used for the visual analyses. The second diagnostic night and the treatment night were classified by two independent scorers into stages. The level of agreement between the 2 scorers was 86.4% (*K* = 0.76). Based on the independent scorings, the consensus sleep staging was formed and used in the statistical analyses. The AHI value was calculated as the number of obstructive apneas and hypopneas (lasting ≥10 s) per hour of sleep. Obstructive apneas were defined as at least a 90% reduction in the thermal signal amplitude, whereas hypopneas were defined as diminution of at least 30% of the nasal pressure signal that was associated with an arousal or desaturation of 3% [24]. Microarousals were scored according to the criteria of the American Sleep Disorders Association [25].

In the diagnostic night, 20 patients had an AHI value of ≥ 10/h (pre-CPAP group). Their sleep parameters and spindle features were compared with the parameters of the age-matched healthy volunteers (control group). After 6 months of CPAP treatment, the patients underwent another polysomnography and the values of that night (CPAP group) were compared with the pre-CPAP values and with the values of the control group.

### 2.2. Spindle Analysis

Spindles were analyzed by applying previously developed automated spindle analysis methods [3, 26]. The main component of these methods is a sigma index that is based on the fast Fourier transform (FFT). The frequency band of 10.5 Hz to 16 Hz was used in computing the sigma index. A high sigma index value indicates a high probability of a spindle and vice versa, and further indicates how dominant the spindle peak is when compared with other EEG activities. Sigma index values over 4.5 generally indicate spindles [26]. The sigma index has built-in artifact rejection functionalities [3], and it can also be complemented with spindle amplitude information [26].

In the present work, the two central (C3-A2, C4-A1) and the two frontopolar (Fp1-A2, Fp2-A1) EEG channels were analyzed separately. Thus, the previous method, described previously [3] needed some minor adjustments. As a result, two EEG channels were processed at a time instead of four. The NREM sleep time was included in the present analysis. As a first step, the procedure detailed in the former work [26] was used to obtain a recording-specific amplitude threshold for the brain region (two EEG channels) by estimating the lower limit of the spindle amplitudes. Then, in order to detect a spindle (from one EEG channel at a time), the sigma index had to exceed 4.5 and also the amplitude threshold had to be exceeded at the same time instant (as in Section 2.2.4 in [3]). The initial spindle detection, obtained at 0.33-s time resolution, was then processed further, and in this way individual unilateral (as in Section  2.2.5 in [3] using m from one EEG channel at a time) and bilateral (as in Section  2.2.6 in [3] with *D* = 1) spindles were identified.

Spindle features were then derived. These features included spindle density (number of spindles per hour of NREM sleep) and median of the intraspindle frequency values (10.5 Hz to 16 Hz, number of cycles per second, in Hz; in bilateral spindles the frequency values were calculated for both hemispheres separately) during NREM sleep. In addition, the bilaterality index (C bil index 1 = the number of bilateral central spindles divided by the number of unilateral C4 spindles (bil C/C4), C bil index 2 = bil C/C3, Fp bil index 1 = bil Fp/Fp2, Fp bil index 2 = bil Fp/Fp1) of the spindles was calculated. If the bilaterality index is <1, the spindles are more often unilateral than bilateral. Finally, the time occupied by spindle sequences (as a percentage of NREM time, at least three consecutive spindles, with interspindle interval ≤ 5 s) was calculated.

As spindle features are found to change dynamically during the night, we calculated all spindle features separately for the first and last part of nocturnal NREM sleep. For this, the NREM time was divided into two. The division was made at the half-way point between the first and last second of the NREM sleep.

The obtained amplitude threshold for the frontopolar spindles was 9.6 *µ*V in the control group (median, range 6.5 to 13.3), 10.6 *µ*V (7.4 to 15.2) in the pre-CPAP group, and 10.0 *µ*V (6.9 to 14.6) in the CPAP group. The respective thresholds for the central spindles were 11.7 *µ*V (median, range 8.5 to 16.6), 11.7 *µ*V (8.2 to 15.4), and 11.1 *µ*V (8.3 to 16.0). The individually determined amplitude thresholds did not differ statistically between the groups.

### 2.3. Statistics

Since some of the parameters were not normally distributed, median and range were used as descriptive statistics and nonparametric tests were chosen in order to compare the study groups. The Mann-Whitney *U* test was used to compare the controls with the patient groups. The Wilcoxon test was used to compare pre-CPAP patients with the CPAP group. To handle the multiple comparisons, appropriate Bonferroni correction factors were used (the original *p* values were multiplied with the number of comparisons). In this way, the significance level was set at 0.05 for all analyses and the reported *p* values are based on two-tailed tests.

## 3. Results

The demographic data and sleep parameters of the study groups are presented in Table 1. The groups did not differ according to age. The OSA patients were more obese than the controls. The controls had more N3 and less N2 than the pre-CPAP group, and they had fewer arousals, desaturations and breathing events than the pre-CPAP group. CPAP treatment decreased the total sleep time of the patients and normalized the number of apneas, arousals, obstructive breathing events and hypoxemic events. However, patients with CPAP continued to show more N2 sleep and less N3 sleep than healthy controls, and their sleep latency was shorter.

### 3.1. Spindle Features in the First Part of the NREM Time

In the first part of the night, neither frontopolar (bil Fp, Fp1, and Fp2) nor central (bil C, C3, and C4) spindle densities differed between the groups (Table 2). In all groups, the Fp- and C- spindles were more often unilateral than bilateral, as revealed by the values of the bilaterality indices (Table 2). There were no statistical differences in the bilaterality indices between the groups. Spindle sequences were few in the first part of the night in all groups and no group-wise differences were found. Hemispheric comparisons revealed that healthy controls had more unilateral central spindles in the left than in the right hemisphere. The pre-CPAP group presented more frontopolar spindles in the left than in the right hemisphere. Other hemispheric spindle density differences were not present (Table 3).

Regarding the intraspindle frequency, all central spindle types (bil C4, bil C3, C4, and C3) were faster than the corresponding frontopolar spindles (bil Fp2, bil Fp1, Fp2, and Fp1, resp.) in all groups (*p* values in Table 4). The frequency of the frontopolar or central spindles of the control group did not differ from the values of the pre-CPAP or CPAP groups (Table 2). The pre-CPAP group had faster Fp2, Fp1, bil C4, bil C3, C4, and C3 spindle frequencies than the CPAP group. Healthy controls did not have hemispheric spindle frequency differences (Table 3). Instead, both the pre-CPAP and CPAP groups presented a minor but significant difference; unilateral frontopolar spindles were faster in the right hemisphere than in the left.

### 3.2. Spindle Features in the Last Part of the NREM Time

In the last part of the night, the control group's bil C and C3 spindle densities were higher than the densities in the pre-CPAP group (Table 5). The control group had more bil C spindles than the CPAP group. The pre-CPAP group had more Fp1 and C4 spindles than the CPAP group. The central spindles of the control group were more often bilateral than in the two patient groups, as assessed by the bilaterality indices. No statistical differences between the groups were found in the spindle sequence times. Healthy controls did not present interhemispheric spindle density differences, but both patient groups had more frontopolar spindles in the right hemisphere than in the left (Table 3).

As in the first part of the NREM time, all central spindle types were faster than the respective frontopolar spindles (Table 4). The spindle frequencies of the control group did not differ from the frequencies of the pre-CPAP or CPAP groups (Table 5). The CPAP group had slower spindles than the pre-CPAP group with the exception of the frequency of the bil Fp1 spindles. Hemispheric spindle frequency differences were not found in any of the groups (Table 3).

### 3.3. Comparison of the Spindle Features between the First and the Last Part of the NREM Time

None of the frontopolar spindle parameters (density, bilaterality index, sequences, and frequency) showed any statistical differences between the first and the last part of the night in any of the study groups.

**Figure 1:**
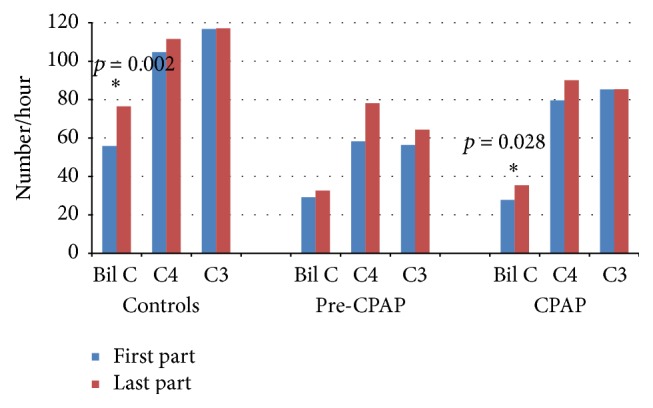
Central spindle densities in the first and the last part of the NREM sleep in the study groups. The statistically significant differences are marked with an asterisk and denoted with the *p* values.

In the control group and the CPAP group, the density of the bilateral central spindles increased towards the end of the night (Figure 1). Instead, unilateral central spindle densities did not show any marked changes during the night in any of the groups.

**Figure 2:**
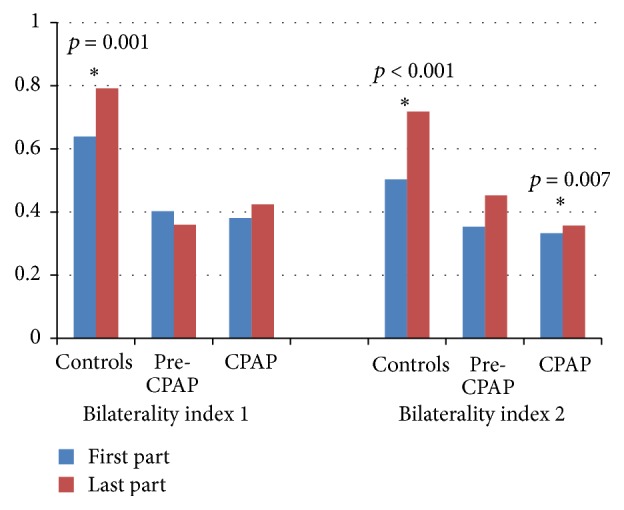
Bilaterality indices of the central spindles in the first and last part of the NREM sleep. The statistically significant differences are marked with an asterisk and denoted with the *p* values.

In the control group, the proportion of bilateral central spindles was higher in the last part of the night than in the first (Figure 2). In the CPAP group, bilaterality index 2 showed an increase in late sleep. The pre-CPAP group did not present significant changes.

**Figure 3:**
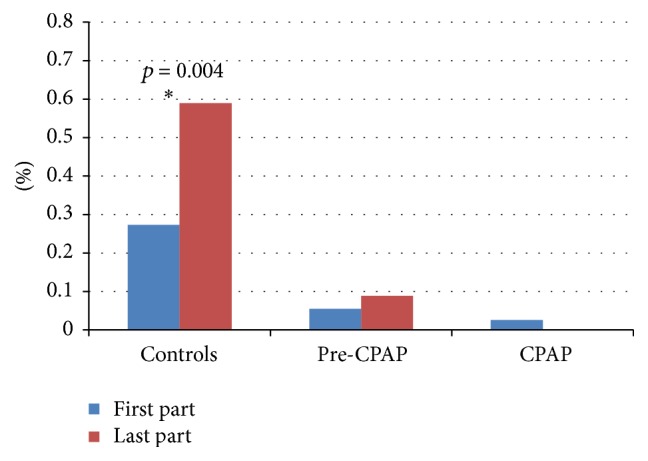
Percentage of time occupied with central spindle sequences in the first and last part of the NREM sleep. The statistically significant differences are marked with asterisk and denoted with the *p* values.

In the controls, the time occupied with central spindle sequences increased towards the end of the night, whereas the sequence time of the patient groups did not change during the night (Figure 3).

**Figure 4:**
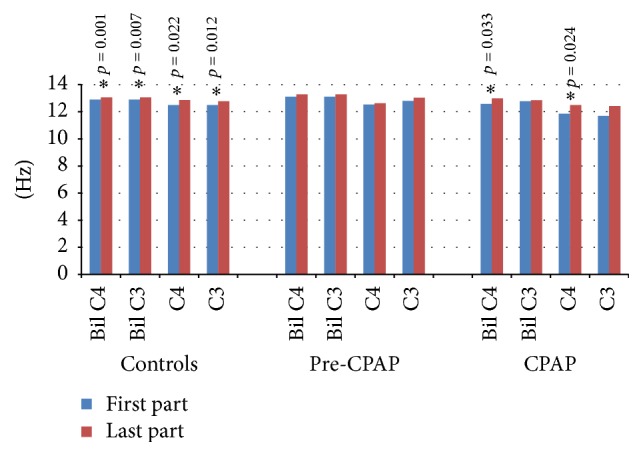
Frequency of the central spindles in the first and last part of the NREM sleep in the study groups. The statistically significant differences are marked with an asterisk and denoted with the *p* values.

In the controls, all central spindle types were faster in the last part of the night than in the first part (Figure 4). The spindle frequencies of the pre-CPAP patients did not change markedly during the night, while both bilateral and unilateral C4 spindles became faster in the CPAP group.

## 4. Discussion

The findings of our study that evaluated sleep spindles after six months of CPAP treatment revealed that many spindle features were normalized by CPAP treatment, but some deficits remained.

Previous studies have shown an increase in spindle density across consecutive NREM sleep episodes in healthy subjects [20, 27–29]. The present results suggest that the density increase is mostly induced by the central, faster spindles but not by frontopolar spindles. This is in line with the findings of Schönwald and coworkers [18]. In contrast to healthy controls, our untreated OSA patients did not present any significant increase in central spindle density during sleep, but with CPAP treatment the increase was recovered.

The increase of central spindles in healthy controls and CPAP-treated patients was restricted to bilateral central spindles only. The density of local central spindles did not show any marked changes during sleep in any of the groups. According to the earlier studies, local spindles are frequent and even more frequent than global spindles [30, 31]. Our results are in line with these findings; bilaterality indices show that the number of unilateral frontopolar and central spindles is higher than bilateral spindles in all groups. However, spindles are found to become more global in late sleep in a small group of neurosurgical patients [30, 31]. Our finding that the proportion of bilateral central spindles increased in late sleep in the healthy controls corresponds with this finding. In OSA patients before CPAP treatment, however, no increase was obtained. CPAP treatment partly normalized this phenomenon as the share of central bilateral spindles increased when compared with left-sided unilateral spindles, but no changes were found in the right hemisphere.

Spindles are abundant in a moderate level of corticothalamic hyperpolarization, in light sleep [32, 33]. With further hyperpolarization, in deep sleep, there are only a few spindles [32, 34–36]. Cortically generated slow oscillation seems to be the principal process behind NREM sleep [32, 37–39] by spreading to the thalamus in synchronous volleys and driving spindles to the cortex through the thalamocortical network [40, 41]. Therefore, we wonder whether the defect in the increasing bilaterality of spindles in OSA would be a consequence of disrupted corticothalamic spindle driving loops. As periodic spindles reflect the periodically recurring spindle driving slow oscillation during light sleep [39], our finding that spindle sequences were not found in OSA patients supports this thought. CPAP treatment did not have an effect on the occurrence of spindle sequences. This might implicate a more permanent defect in the corticothalamic network that disturbs the spindle process.

On the other hand, spindle density has been found to increase locally in those areas of the brain that have been trained during wakefulness in a use-dependent manner [16, 17]. In the present study, the first part of the NREM time did not reveal any spindle density differences between the groups, but local spindles in the left central area (unilateral C3) were more abundant than local spindles in the right central area (unilateral C4) in the controls. Patients with or without CPAP did not present hemispheric differences in the unilateral central spindle densities. In the last part of the NREM time, the density of unilateral left-side central spindles was higher in the control group than in the OSA patients before treatment. It is possible that the left-side abundance in central spindles in healthy controls stems from the finding that the left hemisphere needs more sleep than the right hemisphere, possibly due to differences in daytime activity [42]. If unilateral spindle density is considered use-dependent, then it can be speculated that our control subjects had used their left central cortical areas more than their right ones and that the OSA patients had used their left central brain areas less actively than the controls.

As expected, all frontopolar spindle types were slower than the corresponding central spindles. The frequency of the frontopolar spindles did not change during the night in any of the groups. Instead, all central spindle types became faster towards the morning hours in the controls, whereas no frequency changes were observed in the newly diagnosed OSA patients. CPAP treatment partly normalized the spindle frequencies as bilateral and unilateral right-side central spindles became faster. However, the frequencies of the left-sided central spindles did not change.

The occurrence of spindles has been proven to be dependent on the level of the hyperpolarization in the thalamocortical network, whereas the frequency of spindles has been found to depend on the duration of the hyperpolarization-rebound sequences of the thalamocortical cells [43]. If rebound sequences are long, the spindle is slow. In healthy subjects, these two processes involved in determining spindle features seem to correlate because, in general, spindles are abundant and fast in light sleep and few and slow in deep sleep [9, 33]. However, these processes are presented to be dissociated in OSA patients since slow spindles are found in light sleep [19]. Corresponding results were found in the present study as spindles of the pre-CPAP group did not become faster in late sleep. In addition, the present findings suggest that this dissociation is partly dissolved with CPAP treatment because only left-sided central spindles did not become faster in late sleep with CPAP treatment.

A somewhat surprising finding was that most spindle frequencies were slower with CPAP than without treatment in OSA patients in both the first and the last part of the night. Another interesting finding was that spindle sequences were not recovered with CPAP treatment. However, OSA patients do not usually have deep sleep, which means that thalamocortical hyperpolarization stays at moderate levels. With CPAP, the patients reach slow wave sleep with more negative membrane potentials of the thalamocortical network. This might explain the emergence of slow spindles [9] and a decrease in the number of periodic spindles [10]. In this way, the increase in hyperpolarization would explain both these findings. In addition, it might be that 6 months of CPAP treatment does not abolish the increased sleep pressure of OSA patients properly and increased sleep pressure increases the hyperpolarization level resulting in slower spindles with no periodicity.

The increase in spindle density has been found to be associated with memory function improvement, and it is only the fast spindles that have been associated with memory and learning processing [14, 44–48]. Therefore, we wonder whether the changes in spindle dynamics might be related to the different symptoms of OSA patients such as different cognitive problems [49, 50]. CPAP treatment is known to, at least partially, reduce different symptoms in OSA patients [21, 51], and the fact that many spindle features normalized with CPAP is in line with our hypothesis.

The number and frequency of spindles are dependent on many factors (for review see [33]). Spindles and SWA have a reciprocal relationship [52], and sleep deprivation decreases spindling [53]. In addition, age, gender and circadian rhythm all affect spindle activity [27, 54–56]. In the present study, we studied group-wise differences between healthy controls and OSA patients with and without CPAP treatment, but more importantly we used a within-subject design of OSA patients without and with CPAP treatment to evaluate spindle features. This, in part, reduced the effects of the above mentioned factors that affect the spindle process. A clear limitation of our study is that the assessment of different cognitive domains was not included and future study is needed to evaluate the effect of CPAP treatment on both spindle features and different cognitive skills.

To conclude, CPAP treatment only partially normalizes sleep spindle features. Further studies are therefore needed to evaluate whether these spindle process changes are related to the remaining cognitive problems of OSA patients.

## Figures and Tables

**Table 1:** Median and range of the sleep parameters in the study groups and the *p* values of the comparisons.

	Controls		Pre-CPAP		CPAP		Controls versus pre-CPAP^1^	Controls versus CPAP^1^	Pre-CPAP versus CPAP^2^
	Median	Range	Median	Range	Median	Range	*p* value	*p* value	*p* value
Age	48.5	34–60	50.0	37–65			0.170		
BMI	24.9	20.1–30.2	29.6	24.7–41.4			**<0.001**		
TST min	431.5	348–527	435.8	375–532	395	264–503	0.344	0.256	**0.006**
SEI%	90.6	76.7–97.1	93.2	80.0–97.5	92.0	58.2–97.0	0.417	0.946	0.322
N1%	5.4	2.2–16.3	5.0	1.2–11.2	4.8	1.5–16.1	0.285	0.617	0.490
N2%	60.0	45.4–71.6	73.0	59.5–85.3	68.0	46.8–79.0	**<0.001**	**0.016**	**0.005**
N3%	12.5	2.1–25.4	2.6	0.0–14.1	6.6	0.6–29.3	**<0.001**	**0.029**	**0.002**
REM%	20.0	13.1–27.8	16.8	8.6–27.3	17.6	11.4–26.4	0.117	0.285	0.563
SL min	8.3	3.5–32.5	6.3	1.0–33.0	4.5	2.0–22.0	0.163	**0.014**	0.162
AHI *n*/h	1.8	0.0–4.9	42.0	14.0–103.0	0.7	0.0–14.0	**<0.001**	0.080	**<0.001**
ARI *n*/h	12.8	4.3–22.8	30.0	8.0–99.6	16.0	4.6–27.0	**<0.001**	0.152	**<0.001**
ODI4 *n*/h	0.5	0.0–6.0	34.0	0.0–88.0	0.8	0.0–3.0	**<0.001**	0.496	**<0.001**
Sa02min%	91.0	83.0–95.0	79.0	62.0–92.0	90.5	84.0–94.0	**<0.001**	0.713	**<0.001**
Sa02mean%	95.9	93.0–97.0	93.6	83.1–97.5	96.1	93.3–97.3	**<0.001**	0.551	**<0.001**

^1^Mann-Whitney *U* test; ^2^Wilcoxon test. The Bonferroni corrected *p* values are reported: Bonferroni correction factor = 3.

BMI = body mass index; min = minutes; n/h = number/hour; TST = total sleep time; SEI% = sleep efficiency index; N1%–N3% = time percentage of sleep stages N1–N3 referred to as TST; REM% = time percentage of REM sleep referred to as TST; SL = sleep latency; AHI = apnea/hypopnea index; ARI = arousal index; ODI4 = oxygen desaturation index; Sa02min% = minimum oxygen saturation percentage; Sa02mean% = mean oxygen saturation percentage. Bold font = statistically significant *p* value.

**Table 2:** Median and range of the spindle features in the study groups in the first part of the NREM time.

	Controls		Pre-CPAP		CPAP		Controls versus pre-CPAP^1^	Controls versus CPAP^1^	Pre-CPAP versus CPAP^2^
	Median	Range	Median	Range	Median	Range	*p* value	*p* value	*p* value
Density *n*/h									
Bil Fp dens	33.7	3.4–94.8	22.5	0.3–117.7	23.7	0.0–0.0	0.671	1.0	1.0
Fp2 dens	77.5	14.6–138.6	48.2	8.8–183.9	50.8	0.0–0.0	1.0	1.0	1.0
Fp1 dens	68.0	15.0–129.0	49.0	6.9–143.0	50.9	0.0–0.0	0.912	1.0	1.0
Bil C dens	55.9	11.8–161.2	29.1	1.0–215.7	27.8	1.4–202.8	0.250	0.361	1.0
C4 dens	104.7	36.4–194.2	58.3	13.7–194.3	79.6	16.5–181.5	0.369	1.0	0.741
C3 dens	116.7	50.2–219.6	56.3	17.3–216.3	85.3	29.0–221.5	0.085	0.224	0.574
Frequency Hz									
Bil Fp2 freq	11.8	10.9–12.5	11.5	10.9–13.1	11.4	10.6–12.9	0.702	0.250	0.068
Bil Fp1 freq	11.8	10.9–12.9	11.5	10.7–13.1	11.5	10.8–12.8	0.874	0.516	1.0
Fp2 freq	11.8	10.9–12.3	11.5	10.8–13.1	11.5	10.8–12.8	1.0	1.0	**0.013**
Fp 1 freq	11.8	11.0–12.5	11.5	10.9–13.0	11.4	10.9–12.8	0.893	0.272	**0.042**
Bil C4 freq	12.9	11.2–14.4	13.1	11.0–14.6	12.6	10.7–14.0	1.0	0.703	**0.006**
Bil C3 freq	12.9	11.1–14.3	13.1	11.3–14.9	12.8	10.9–14.4	0.670	1.0	**0.007**
C4 freq	12.5	11.2–13.7	12.5	11.0–14.2	11.9	11.0–13.8	1.0	0.283	**0.043**
C3 freq	12.5	11.1–13.8	12.8	11.3–15.1	11.7	11.0–13.9	1.0	0.207	**0.001**
Sequences %									
Fp sequences	0.0	0.0–0.4	0.0	0.0–1.1	0.0	0.0–0.3	1.0	1.0	0.999
C sequences	0.3	0.0–2.4	0.1	0.0	4.4	0.0–3.1	0.720	0.176	0.224
Bilaterality indices									
Fp bil index 1	0.48	0.21–0.98	0.36	0.02–0.81	0.44	0.07–0.68	0.185	0.388	1.0
Fp bil index 2	0.54	0.21–0.89	0.36	0.04–1.18	0.44	0.06–0.95	0.388	0.275	1.0
C bil index 1	0.64	0.20–1.31	0.40	0.03–1.16	0.38	0.08–1.12	0.297	0.085	1.0
C bil index 2	0.50	0.14–1.13	0.35	0.06–1.0	0.33	0.05–0.98	0.611	0.145	1.0

^1^Mann-Whitney *U* test; ^2^Wilcoxon test. The Bonferroni corrected *p* values are reported: Bonferroni correction factor = 3.

Bil Fp/bil C = bilateral frontopolar/central spindle; bil C4, bil C3 = bilateral right-side and left-side spindles, respectively; Fp2, Fp1, C4, and C3 = unilateral spindles; sequences = percentage of time referred to as TST occupied by periodic frontopolar or central spindles; Fp bil index 1 = number of bil Fp spindles divided by the number of unilat Fp2 spindles (bil Fp/Fp2); Fp bil index 2 = number of bil Fp spindles divided by the number of unilat Fp1 spindles (bil Fp/Fp1); C bil index 1 = number of bil C spindles divided by the number of unilat C4 spindles (bil C/C4); C bil index 2 = number of bil C spindles divided by the number of unilat C3 spindles (bil C/C3).

**Table 3:** *p* values of the hemispheric right-left comparisons of the bilateral and unilateral spindle frequencies and unilateral densities in the different study groups.

	Controls	Pre-CPAP	CPAP
*First part of the NREM time*			
Density			
Fp2 versus Fp1	0.575	**0.044**	0.550
C4 versus C3	**0.030**	0.737	0.156
Frequency			
Bil Fp2 versus bil Fp1	0.717	0.809	0.070
Fp2 versus Fp1	0.126	**0.035**	**0.014**
Bil C4 versus bil C3	0.588	0.235	0.064
C4 versus C3	0.173	0.145	0.794
*Last part of the NREM time*			
Density			
Fp2 versus Fp1	0.601	**0.006**	**0.048**
C4 versus C3	0.469	0.478	0.502
Frequency			
Bil Fp2 versus bil Fp1	0.717	0.658	0.225
Fp2 versus Fp1	0.444	0.225	0.313
Bil C4 versus bil C3	0.444	0.097	0.896
C4 versus C3	0.365	0.082	0.911

Numbers refer to *p* values based on the Wilcoxon test.

**Table 4:** *p* values of the comparisons between the central and frontopolar spindle frequencies in the different study groups.

	Controls	Pre-CPAP	CPAP
First part of the NREM time			
Bil C4 versus bil Fp2	<0.001	<0.001	0.003
Bil C3 versus bil Fp1	<0.001	<0.001	0.003
C4 versus Fp2	<0.001	0.001	0.022
C3 versus Fp1	<0.001	<0.001	0.002
Last part of the NREM time			
Bil C4 versus bil Fp2	<0.001	0.002	<0.001
Bil C3 versus bil Fp1	<0.001	<0.001	<0.001
C4 versus Fp2	<0.001	0.001	0.001
C3 versus Fp1	<0.001	<0.001	<0.001

Numbers refer to *p* values based on the Wilcoxon test.

**Table 5:** Median and range of the spindle features in the study groups at the last part of the NREM time.

	Controls		Pre-CPAP		CPAP		Controls versus pre-CPAP^1^	Controls versus CPAP^1^	Pre-CPAP versus CPAP^2^
	Median	Range	Median	Range	Median	Range	*p* value	*p* value	*p* value
Density *n*/h									
Bil Fp dens	43.7	7.3–110.3	23.2	0.5–105.7	23.6	1.1–102.6	0.350	0.533	1.0
Fp2 dens	85.1	26.6–136.9	52.5	10.6–157-7	54.4	10.1–159.3	0.768	1.0	0.741
Fp1 dens	65.6	15.9–150.5	43.8	7.1–141.3	46.4	12.7–129.0	0.223	0.518	**0.033**
Bil C dens	76.5	18.3–215.7	32.6	0.5–282.5	35.4	4.4–282.0	**0.010**	**0.018**	0.436
C4 dens	111.5	63.8–183.5	78.2	21.6–196.8	90.1	18.6–191.8	0.223	0.626	**0.027**
C3 dens	117.1	46.4–229.7	64.3	20.2–231.6	85.4	26.9–217.7	**0.033**	0.093	0.238
Frequency Hz									
Bil Fp2 freq	11.7	11.1–12.4	11.6	11.0–13.6	11.3	10.8–13.0	1.0	0.074	**0.001**
Bil Fp1 freq	11.8	11.1–12.4	11.6	10.8–13.3	11.4	10.7–13.0	1.0	0.243	0.131
Fp2 freq	11.7	11.2–12.4	11.7	10.9–13.0	11.5	11.0–12.8	1.0	0.395	**0.022**
Fp 1 freq	11.9	11.2–12.4	11.6	11.1–13.0	11.4	11.0–12.9	0.640	0.159	**0.037**
Bil C4 freq	13.1	11.4–14.3	13.3	10.8–14.9	13.0	10.9–14.6	1.0	1.0	**0.007**
Bil C3 freq	13.1	11.2–14.3	13.3	10.8–14.8	12.8	10.9–14.8	0.912	1.0	**0.004**
C4 freq	12.9	11.2–14.1	12.6	11.0–14.5	12.5	10.9–13.9	1.0	1.0	**0.041**
C3 freq	12.8	11.1–14.3	13.0	10.9–15.0	12.4	10.9–13.9	1.0	1.0	**0.005**
Sequences %									
Fp sequences	0.0	0.0–0.5	0.0	0.0–0.5	0.0	0.0–0.5	1.355	1.0	0.999
C sequences	0.6	0.0–4.0	0.1	0.0–7.3	0.0	0.0–7.1	0.086	0.097	1.0
Bilaterality indices									
Fp bil index 1	0.47	0.15–1.22	0.36	0.04–0.73	0.43	0.11–0.75	0.168	0.388	1.0
Fp bil index 2	0.61	0.15–1.07	0.48	0.04–1.15	0.46	0.09–1.05	0.506	0.191	1.0
C bil index 1	0.79	0.17–1.55	0.36	0.03–1.44	0.42	0.14–1.47	**0.006**	**0.008**	1.0
C bil index 2	0.72	0.20–1.38	0.45	0.03–1.22	0.36	0.15–1.30	**0.015**	**0.009**	1.0

^1^Mann-Whitney *U* test; ^2^Wilcoxon test. The Bonferroni corrected *p* values are reported: Bonferroni correction factor = 3.

Abbreviations as in Table 2.

## Additional Points

*Highlights*. (i) Obstructive sleep apnea distorts sleep spindle process. (ii) CPAP treatment normalizes many spindle features but some deficits remain. (iii) The remaining spindle changes might be related to cognitive disorders of CPAP-treated patients.
